# Task difficulty affects the predictive process indexed by visual mismatch negativity

**DOI:** 10.3389/fnhum.2013.00267

**Published:** 2013-06-12

**Authors:** Motohiro Kimura, Yuji Takeda

**Affiliations:** Cognition and Action Research Group, Human Technology Research Institute, National Institute of Advanced Industrial Science and TechnologyTsukuba, Japan

**Keywords:** attention, event-related brain potential, perceptual load, predictive process, prediction error, task difficulty, visual mismatch negativity

## Abstract

Visual mismatch negativity (MMN) is an event-related brain potential (ERP) component that is elicited by prediction-incongruent events in successive visual stimulation. Previous oddball studies have shown that visual MMN in response to task-irrelevant deviant stimuli is insensitive to the manipulation of task difficulty, which supports the notion that visual MMN reflects attention-independent predictive processes. In these studies, however, visual MMN was evaluated in deviant-minus-standard difference waves, which may lead to an underestimation of the effects of task difficulty due to the possible superposition of N1-difference reflecting refractory effects. In the present study, we investigated the effects of task difficulty on visual MMN, less contaminated by N1-difference. While the participant performed a size-change detection task regarding a continuously-presented central fixation circle, we presented oddball sequences consisting of deviant and standard bar stimuli with different orientations (9.1 and 90.9%) and equiprobable sequences consisting of 11 types of control bar stimuli with different orientations (9.1% each) at the surrounding visual fields. Task difficulty was manipulated by varying the magnitude of the size-change. We found that the peak latencies of visual MMN evaluated in the deviant-minus-control difference waves were delayed as a function of task difficulty. Therefore, in contrast to the previous understanding, the present findings support the notion that visual MMN is associated with attention-demanding predictive processes.

## Introduction

### Predictive processes indexed by visual mismatch negativity

The ability to extract sequential rules embedded in the temporal structure of sensory events and to predict upcoming sensory events based on the extracted sequential rules is crucial for successful adaptation to the external environment (e.g., Mumford, 1992; Friston, 2003, 2005). Recent electrophysiological studies have shown that such predictive processes in vision are well reflected by visual mismatch negativity (MMN), an event-related brain potential (ERP) component (for reviews, see Pazo-Alvarez et al., 2003; Czigler, 2007; Kimura et al., 2011; Kimura, 2012; Winkler and Czigler, 2012). Visual MMN is a negative-going ERP component with a posterior scalp distribution that usually emerges at around 150–400 ms after the onset of visual events. This component has been most typically observed in response to infrequent deviant stimuli that are randomly inserted among frequent standard stimuli (i.e., an oddball sequence). Importantly, however, the elicitation of visual MMN is not limited to such physically deviant stimuli, but rather includes a variety of stimuli that violate concrete or abstract sequential rules (e.g., Czigler et al., 2006; Kimura et al., 2010b, 2012; Stefanics et al., 2011). This leads to the notion that visual MMN emerges when a current visual event is incongruent with visual events that are predicted on the basis of extracted sequential rules (i.e., prediction error account of visual MMN; Kimura et al., 2011; Kimura, 2012).

### Attention-independent predictive processes

One of the unique aspects of visual MMN elicitation is its automaticity. In most previous studies, visual MMN has been observed in response to deviant stimuli when oddball sequences are unrelated to the task and are not actively attended by the participant. This indicates that the elicitation of visual MMN is largely automatic and obligatory. This notion is further strengthened by the finding that visual MMN elicited by task-irrelevant deviant stimuli is insensitive to the manipulation of task difficulty (Heslenfeld, 2003; Pazo-Alvarez et al., 2004). Heslenfeld (2003) presented task-irrelevant oddball sequences consisting of deviant and standard grating stimuli with different spatial frequencies at the peripheral visual fields while the participant performed a visuo-motor tracking task that involved a small, continuously moving rectangle presented at the central visual field. The difficulty of the tracking task was manipulated among three levels (easy, moderate, and difficult) by varying the speed and frequency of changes in direction of the moving rectangle. Visual MMN elicited by deviant stimuli did not differ as a function of task difficulty. Pazo-Alvarez et al. (2004) obtained similar results. They presented task-irrelevant oddball sequences consisting of deviant and standard grating stimuli with different directions of motion at the peripheral visual fields while the participant performed a discrimination task that involved small colored digits discretely presented at the central visual field. The difficulty of the discrimination task was manipulated between two levels (easy and difficult) by asking the participant to perform either a task that involved the digit numbers (easy) or a task that involved the combination of both the digit numbers and the color of digits (difficult). Visual MMN elicited by deviant stimuli did not differ between the two task-difficulty conditions. According to the perceptual load theory of attention (Lavie and Tsal, 1994; Lavie, 1995, 2005), the task difficulty in the perceptual discrimination (i.e., perceptual load) of task-relevant information is one of the critical factors that determine the amount of attentional allocation to peripherally presented task-irrelevant information. Therefore, the lack of a task difficulty effect suggests that visual MMN is insensitive to the amount of attentional allocation, which leads to the notion that visual MMN reflects attention-independent predictive processes.

### Present study

**Figure 1:**
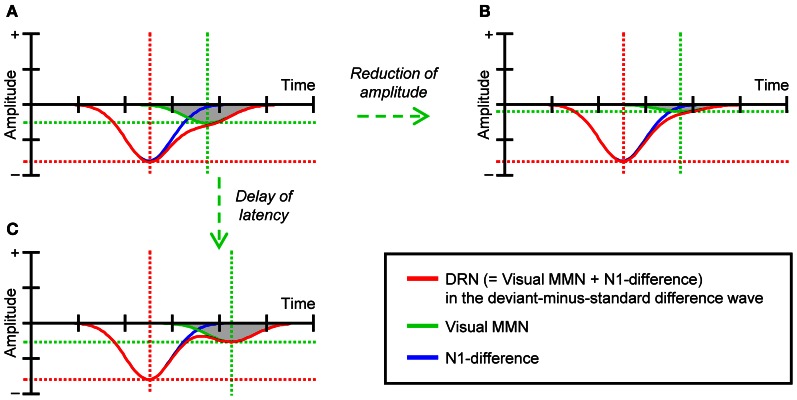
**(A)** A schematic illustration of DRN, visual MMN, and N1-difference. **(B)** The modeled DRN as the sum of visual MMN with reduced amplitudes and N1-difference. **(C)** The modeled DRN as the sum of visual MMN with delayed latencies and N1-difference.

Although the results described by Heslenfeld (2003) and Pazo-Alvarez et al. (2004) support the notion that attention-independent predictive processes underlie visual MMN, this idea needs to be studied further. In these previous studies, visual MMN was evaluated by comparing ERPs elicited by infrequent deviant stimuli to those elicited by frequent standard stimuli (i.e., deviant-minus-standard difference waves). However, more recent studies have questioned the validity of this comparison for the evaluation of visual MMN (see e.g., Czigler, 2007; Kimura et al., 2011; Kimura, 2012). This is because, due to the large difference in probability between deviant and standard stimuli, the state of refractoriness (or the level of habituation) of afferent neurons that specifically respond to the feature value of deviant stimuli can be drastically lower than that of afferent neurons that specifically respond to the feature value of standard stimuli. In other words, the amplitudes of visual evoked potentials (in particular, N1) in response to deviant stimuli can be substantially greater than those of N1 in response to standard stimuli. As a result, the classical visual MMN extracted in deviant-minus-standard difference waves [we refer to this effect as deviant-related negativity (DRN)] can include not only visual MMN elicited by deviant stimuli (i.e., prediction error effects) but also the N1-difference between deviant and standard stimuli (i.e., refractory effects) (for a more detailed discussion, see e.g., Czigler et al., 2002; Kenemans et al., 2003; Kimura et al., 2009). If we consider that these two effects often overlap each other both spatially and temporally in deviant-minus-standard difference waves (see e.g., Maekawa et al., 2005; Kimura et al., 2009, 2010b), it is possible that the effects of task difficulty on visual MMN have been underestimated in previous studies (Heslenfeld, 2003; Pazo-Alvarez et al., 2004). For example, if we assume that visual MMN and N1-difference contribute to DRN, as illustrated in Figure 1A (for similar empirical data, see e.g., Kimura et al., 2009, 2010b), neither the reduction of amplitudes (Figure 1B) nor the delay of latencies of visual MMN (Figure 1C) may be detected, at least with common ERP analyses that focus on the peak of DRN.

**Figure 2:**
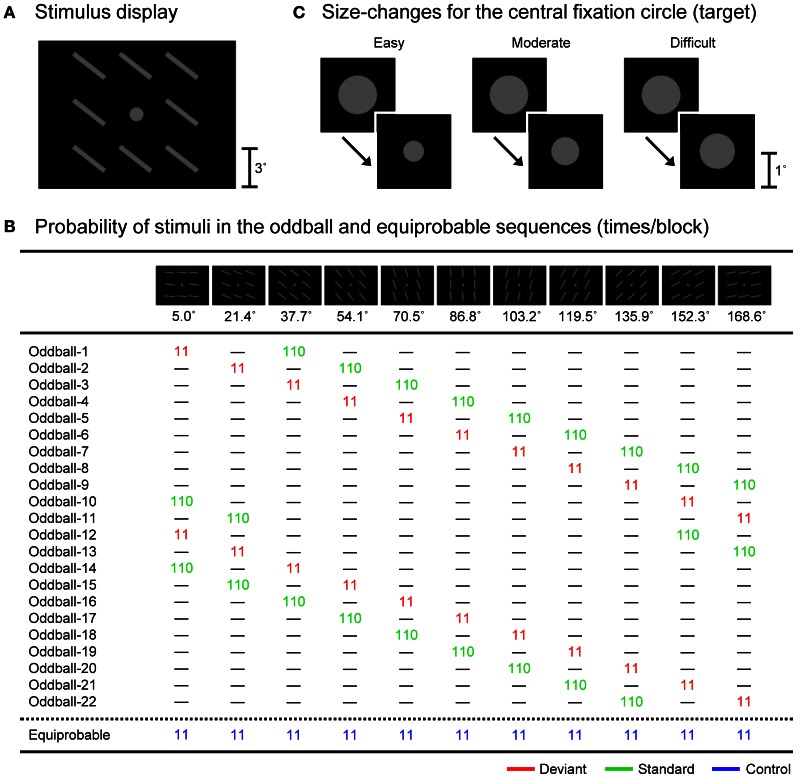
**(A)** An example of the stimulus display. **(B)** Stimuli and their probabilities (times/block) in the oddball and equiprobable sequences. **(C)** Size-changes for the central fixation circle in three task-difficulty conditions (easy, moderate, and difficult).

By considering this possibility, in the present study, we examined the effects of task difficulty on visual MMN, less contaminated by N1-difference. We used a so-called “equiprobable” protocol that allows for the reliable dissociation of visual MMN and N1-difference (e.g., Kimura et al., 2009; for the original protocol, see Schröger and Wolff, 1996; Schröger, 1997; Jacobsen and Schröger, 2001). While the participant performed a size-change detection task for a small fixation circle that was continuously presented at the central visual field, we presented either (1) typical oddball sequences consisting of the randomized presentation of deviant and standard bar stimuli with different orientations (e.g., 5.0 and 37.7° to the right from the horizontal; 9.1 and 90.9%, respectively) or (2) equiprobable sequences consisting of the randomized presentation of 11 types of equiprobable control bar stimuli with different orientations (5.0, 21.4, 37.7, 54.1, 70.5, 86.8, 103.2, 119.5, 135.9, 152.3, and 168.6°; 9.1% each) at the surrounding visual fields in separate blocks (see Figure 2B). In this protocol, while the deviant stimuli should elicit visual MMN, the standard and control stimuli should not. This is because the standard and control stimuli do not violate any sequential rule. In addition, N1 elicited by the deviant stimuli should be equal to (or even smaller than) N1 elicited by the control stimuli, and should be greater than N1 elicited by the standard stimuli. This is because the probability of the deviant and control stimuli is kept the same (9.1%) and is lower than the probability of the standard stimuli (90.9%), and further, the physical separation among control stimuli (ca. 45.0°, on average) is kept greater than that between deviant and standard stimuli (ca. 32.7°) (for more detailed information, see Schröger and Wolff, 1996; Schröger, 1997; Jacobsen and Schröger, 2001; Kimura et al., 2009, 2010b). Thus, visual MMN (and possibly a small polarity-reversed N1-difference) should be extracted by comparing ERPs elicited by the deviant stimuli to those elicited by the control stimuli (i.e., deviant-minus-control difference waves), while N1-difference should be extracted by comparing ERPs elicited by the control stimuli to those elicited by the standard stimuli (i.e., control-minus-standard difference waves). The difficulty of the size-change detection task for the central fixation circle was manipulated among three levels (easy, moderate, and difficult) by varying the magnitude of the size-change. With this experimental design, we examined the effects of task difficulty on visual MMN as well as DRN and N1-difference, and investigated whether or not the predictive processes reflected by visual MMN are truly attention-independent.

## Methods

### Participants

Twenty-two undergraduate and graduate university students (7 women, 15 men; age range = 20–25 years, mean = 21.5 years) participated in this experiment. Twenty-one participants were right-handed and one was left-handed. All participants had normal or corrected-to-normal vision and were free of neurological or psychiatric disorders. Written informed consent was obtained from each participant after the nature of the study had been explained. The experiment was approved by the National Institute of Advanced Industrial Science and Technology (AIST) Safety and Ethics committee.

### Stimuli and procedure

All stimuli were presented on a 17-inch cathode ray tube (CRT) display (Sony, Trinitron Multiscan G220), which was controlled by programs written in MATLAB (Mathworks, Inc.) with the Psychophysics Toolbox (Brainard, 1997; Pelli, 1997) installed on a computer (Apple, MacPro 1,1; NVIDIA, GeForce 7300GT). Figure 2A shows an example of the stimulus display consisting of a central fixation circle and surrounding bars. Eleven types of bar stimuli were used (Figure 2B). Each bar stimulus consisted of eight gray bars (luminance of 14.5 cd/m^2^ and visual angle of 3.0° (length) × 0.4° (width) from a viewing distance of 70 cm, respectively) at eight surrounding locations (3.3° upper, lower, left, and right and 4.6° upper-left, upper-right, lower-left, and lower-right from the center of the display to the center of each bar, respectively) against a black background (luminance of 0.1 cd/m^2^). The 11 types of surrounding bar stimuli differed in the orientation of the bars (5.0, 21.4, 37.7, 54.1, 70.5, 86.8, 103.2, 119.5, 135.9, 152.3, and 168.6° to the right from the horizontal (ca. 16.4° step), respectively). The exposure duration of the surrounding bar stimuli was fixed at 250 ms and the stimulus onset asynchrony was fixed at 500 ms (i.e., the inter-stimulus interval was fixed at 250 ms) in all conditions.

These surrounding bar stimuli were presented in 23 types of stimulus sequences (Figure 2B): 22 oddball sequences and one equiprobable sequence. In the oddball sequences, two types of surrounding bar stimuli (deviant and standard, 11 and 110 times/block, i.e., 9.1 and 90.9%, respectively) were presented in random order, with the exception that a standard stimulus was presented at least 11 times at the beginning of each block and each deviant stimulus was followed by at least one standard stimulus. In the equiprobable sequences, 11 types of surrounding bar stimuli (control, 11 times/block each, i.e., 9.1% each) were presented in random order, with the exception that each control stimulus was followed by at least one control stimulus with a different orientation. Through the use of these stimulus sequences, we could ensure that, on average, the physical properties of deviant, standard, and control stimuli were the same, which allowed us to evaluate visual MMN as well as N1-difference and DRN without any contamination by the effects of physical differences in the eliciting stimuli. Also, in these stimulus sequences, the probability of control stimuli was kept the same as that of deviant stimuli (9.1%), and the physical separation among control stimuli (ca. 45.0°, on average) was kept greater than that between deviant and standard stimuli (ca. 32.7°), which guaranteed that the state of refractoriness for control stimuli was equal to (or may be even lower than) that for deviant stimuli.

In addition to the surrounding bar stimuli, a gray fixation circle (luminance of 14.5 cd/m^2^ and visual angle of 1.1 × 1.1°) was continuously presented at the center of the display throughout the blocks (Figure 2A). From time to time, the size of the fixation circle suddenly became smaller. The mean frequency of the size-change was four times/block (ranging from three to five times/block) and the exposure duration of the size-changed fixation circle was 100 ms in all conditions. To ensure that the timing of the size-change was independent of the surrounding bar stimulation, we segmented the whole period of each block into consecutive 50-ms intervals and randomly selected two consecutive intervals for the size-change (i.e., 100 ms), with the exception that the size-change did not occur within the 5.5-s interval at the beginning of each block (where the first 11 surrounding bar stimuli were presented) and at least a 1.5-s interval was inserted between a size-change and the subsequent size-change. To manipulate the task difficulty, three levels of magnitude of the size-change were used in separate blocks (Figure 2C): from 1.1 × 1.1° to 0.6 × 0.6° in the easy condition, to 0.8 × 0.8° in the moderate condition, and to 1.0 × 1.0° in the difficult condition.

The experiment consisted of 66 blocks (11 blocks for the easy oddball condition, 11 blocks for the moderate oddball condition, 11 blocks for the difficult oddball condition, 11 blocks for the easy equiprobable condition, 11 blocks for the moderate equiprobable condition, and 11 blocks for the difficult equiprobable condition), each of which consisted of the presentation of 121 surrounding bar stimuli. For half of the participants, oddball sequences #1–11 and the equiprobable sequence (Figure 2B) were used, while for the other half of the participants, oddball sequences #12–22 and the equiprobable sequence (Figure 2B) were used. The order of these blocks was randomized across participants.

The participant was seated in a reclining chair in a sound-attenuated and electrically-shielded dimly lit room. Before the start of the experiment, the participant was instructed to focus on a fixation circle, ignore surrounding bars, and press a button with the right index finger as quickly and accurately as possible when the fixation circle became smaller. The participant was also asked to minimize any eye movement and blinking during each block. Before the start of each block, the participant was informed about the magnitude of the size-change of the fixation circle in the upcoming block (i.e., large, medium, or small).

### Recordings

The electroencephalogram (EEG) was recorded with a digital amplifier (Nihon-Kohden, Neurofax EEG1100) and silver-silver chloride electrodes placed at 26 scalp sites (Fp1, Fp2, F7, F3, Fz, F4, F8, FCz, T3, C3, Cz, C4, T4, T5, P3, Pz, P4, T6, PO7, PO3, POz, PO4, PO8, O1, Oz, and O2 according to the extended International 10–20 System). All electrodes were referenced to the nose tip. To monitor blinks and eye movements, vertical and horizontal electrooculograms (EOGs) were also recorded with two electrodes above and below the right eye and two electrodes at the right and left outer canthi of the eyes, respectively. The impedance of all electrodes was kept below 10 kΩ. The EEG and EOG signals were digitized at a sampling rate of 1000 Hz and bandpass-filtered at 1–30 Hz with a finite impulse response (FIR) filter. The EEG and EOG signals time-locked to the onset of surrounding bar stimuli were then averaged for nine categories defined by three stimulus types (deviant, standard, and control) and 3 task difficulties (easy, moderate, and difficult). Averaging epochs were 600 ms featuring a 100-ms pre-stimulus baseline. In the averaging procedure, (1) the first three epochs in each block, (2) epochs during which the size-change of the fixation circle occurred and the two subsequent epochs, (3) epochs during which the participant made a button press and the two subsequent epochs, (4) epochs preceded by deviant stimuli, and (5) epochs in which the signal changes exceeded ± 80 μV on any of the electrodes, were excluded. As a result, the averaging number for deviant, standard, and control stimuli was, on average, 89, 735, and 921 times for the easy condition, 89, 735, and 919 times for the moderate condition, and 91, 745, and 928 times for the difficult condition, respectively.

### Data analysis

#### Behavioral performance

Behavioral performance was measured in terms of reaction time (ms), hit rate (%), and false alarm (times/block). Responses were scored as a hit if the button was pressed within 200–1000 ms after the onset of the change in the fixation circle. Responses outside this period were classified as a false alarm. These measures were subjected to repeated-measures ANOVAs with two factors: 2 Sequences (Oddball vs. Equiprobable) and 3 Task difficulties (Easy, Moderate, vs. Difficult). The Greenhouse–Geisser ε correction for the violation of sphericity was applied when appropriate. Effect sizes were calculated as partial eta squared (η^2^). *Post-hoc* comparisons involved paired *t*-tests with the Bonferroni correction.

#### ERPs and difference waves

Grand-average deviant-minus-standard difference waves were calculated for the three task-difficulty conditions. In the difference waves, a bilateral parieto-occipital (PO7 and PO8) maximum negativity (DRN) that peaked at 188 ms (easy condition, PO8), 196 ms (moderate condition, PO8), and 203 ms (difficult condition, PO8) was observed. To decompose DRN into N1-difference and visual MMN (and possibly, small polarity-reversed N1-difference), grand-average control-minus-standard and deviant-minus-control difference waves were then calculated for the three task-difficulty conditions, respectively. In the control-minus-standard difference waves, a bilateral parieto-occipital (PO7 and PO8) maximum negativity (N1-difference) that peaked at 193 ms (easy condition, PO8), 196 ms (moderate condition, PO8), and 197 ms (difficult condition, PO8) was observed. In the deviant-minus-control difference waves, a right parieto-occipital (PO8) maximum negativity (visual MMN) that peaked at 186 ms (easy condition, PO8), 193 ms (moderate condition, PO8), and 225 ms (difficult condition, PO8) was observed.

#### Scalp distributions of N1-difference and visual MMN

To compare the scalp distributions of N1-difference and visual MMN, the mean amplitudes of the control-minus-standard and deviant-minus-standard difference waves (within the 11-ms time-windows including ± 5 ms from the corresponding peak) at 13 posterior electrodes in the three task-difficulty conditions were subjected to repeated-measures ANOVAs with three factors: 2 Difference waves (Control-minus-standard vs. Deviant-minus-control), 13 Electrodes (T5, P3, Pz, P4, T6, PO7, PO3, POz, PO4, PO8, O1, Oz, vs. O2), and 3 Task difficulties (Easy, Moderate, vs. Difficult). Further, the same analysis was performed on the amplitude values that were normalized by vector length, where, for each of the six conditions defined by two difference waves and three task-difficulty conditions, each amplitude value was divided by the square root of the sum of the squared amplitudes over the 13 electrode locations (McCarthy and Wood, 1985). The Greenhouse–Geisser ε correction for the violation of sphericity was applied when appropriate. Effect sizes were calculated as partial η^2^. *Post-hoc* comparisons involved paired *t*-tests with the Bonferroni correction.

#### Mean amplitudes of DRN, N1-difference, and visual MMN

To test the significance of the elicitation of DRN, N1-difference, and visual MMN, the mean amplitudes of the deviant-minus-standard, control-minus-standard, and deviant-minus-control difference waves (within the 11-ms time-windows including ± 5 ms from the corresponding peak) at an electrode (PO8, where these components had the maximum amplitudes) in the three task-difficulty conditions were subjected to one-tailed paired *t*-tests. The effect sizes are presented as *d*-values. Further, to compare the mean amplitudes of each component among the three task-difficulty conditions, the mean amplitudes of each component were subjected to repeated-measures ANOVAs with one factor: 3 Task difficulties (Easy, Moderate, vs. Difficult). The Greenhouse–Geisser ε correction for the violation of sphericity was applied. Effect sizes were calculated as partial η^2^. *Post-hoc* comparisons involved paired *t*-tests with the Bonferroni correction.

#### Peak latencies of DRN, N1-difference, and visual MMN

To estimate the peak latencies of DRN, N1-difference, and visual MMN, a jackknife method was used (Miller et al., 1998; Ulrich and Miller, 2001; Kiesel et al., 2008). With regard to 22 sub-grand-average difference waves at PO8 electrode for each component in each task-difficulty condition, the peak latency was evaluated as the time at which the difference waves reached the peak amplitude of each component, within 100–300 ms after stimulus onset. To compare the peak latencies of each component among the three task-difficulty conditions, the evaluated peak latencies of each component were then subjected to repeated-measures ANOVAs with one factor: 3 Task difficulties (Easy, Moderate, vs. Difficult). The Greenhouse–Geisser ε correction for the violation of sphericity was applied. The *F*-values were corrected according to Ulrich and Miller (2001). The effect sizes are shown as partial η^2^. *Post-hoc* comparisons involved paired *t*-tests with the Bonferroni correction.

#### Mean amplitudes of visual evoked potentials

To examine the effects of task difficulty on visual evoked potentials, the mean amplitudes of standard and control ERPs (within each of 20 consecutive 10-ms time-windows from 100 to 300 ms) at the PO8 electrode in the three task-difficulty conditions were subjected to repeated-measures ANOVAs with two factors: 2 Stimuli (Standard vs. Control) and 3 Task difficulties (Easy, Moderate, vs. Difficult). The Greenhouse–Geisser ε correction for the violation of sphericity was applied. Effect sizes were calculated as partial η^2^. *Post-hoc* comparisons involved paired *t*-tests with the Bonferroni correction.

## Results

### Behavioral performance

The mean reaction time in the oddball condition was 459 ms (*SD* = 79), 463 ms (77), and 480 ms (77), while that in the equiprobable condition was 459 ms (78), 467 ms (76), and 479 ms (79), in the easy, moderate, and difficult conditions, respectively. Two-Way ANOVAs (2 Sequences × 3 Task difficulties) revealed a significant main effect of Task difficulty [*F*_(2, 42)_ = 11.49, *p* < 0.001, ε = 0.97, partial η^2^ = 0.35]. *Post-hoc* comparisons showed that the reaction time in the difficult condition was longer than those in both the easy (*p* < 0.001) and moderate conditions (*p* < 0.05). The hit rate in the oddball condition was 95.4% (*SD* = 5.3), 94.5% (6.9), and 88.4% (11.9), while that in the equiprobable condition was 95.3% (6.3), 93.4% (8.6), and 87.6% (10.9), in the easy, moderate, and difficult conditions, respectively. Two-Way ANOVAs (2 Sequences × 3 Task difficulties) revealed a significant main effect of Task difficulty [*F*_(2, 42)_ = 21.24, *p* < 0.001, ε = 0.74, partial η^2^ = 0.50]. *Post-hoc* comparisons showed that the hit rate in the difficult condition was lower than those in both the easy (*p* < 0.001) and moderate conditions (*p* < 0.01). The false alarm was negligible in all conditions (on average, less than 0.1 times/block). Two-Way ANOVAs (2 Sequences × 3 Task difficulties) revealed no significant effects (*F*s < 1.0).

### ERPs and difference waves

**Figure 3:**
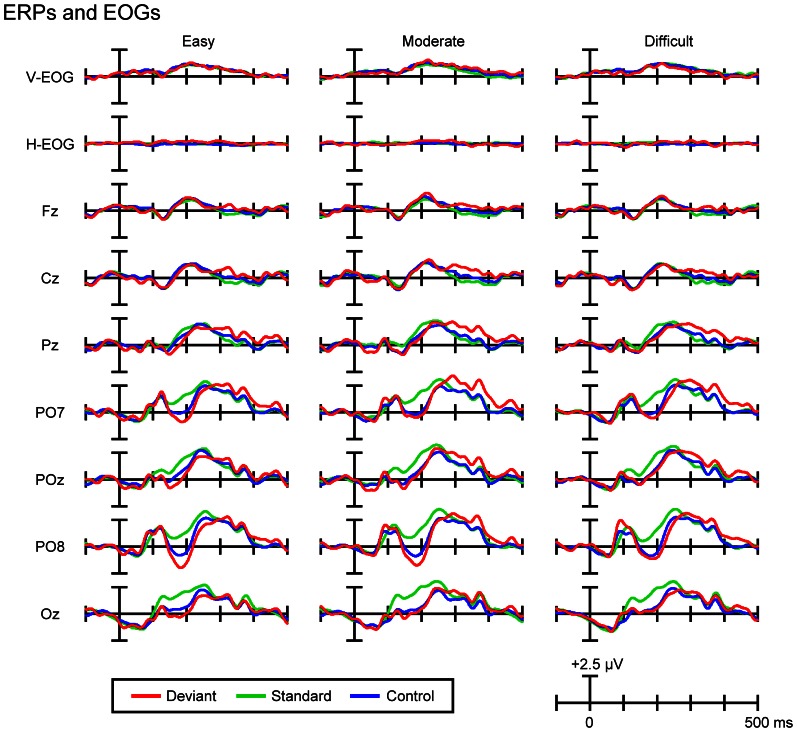
**Grand-average ERPs and EOGs in response to deviant, standard, and control stimuli in the easy (left column), moderate (middle column), and difficult conditions (right column)**.

**Figure 4:**
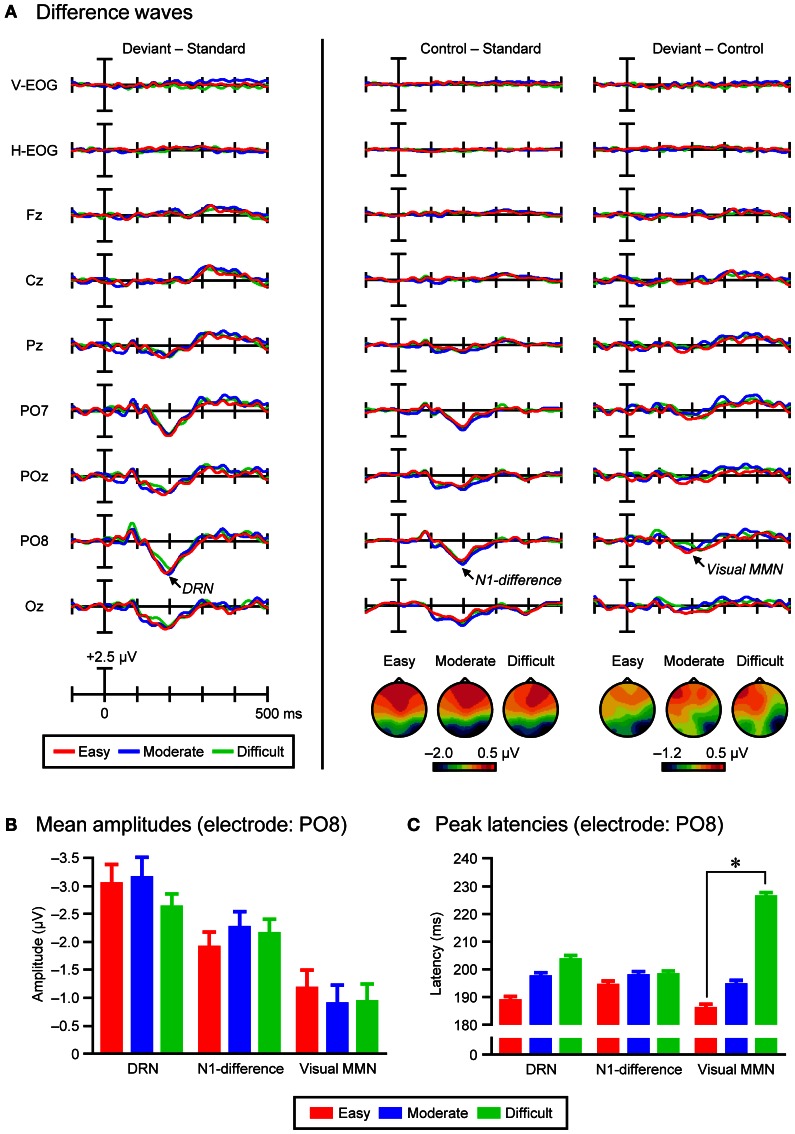
**(A)** Grand-average deviant-minus-standard difference waves (left column), grand-average control-minus-standard difference waves and topographical maps of N1-difference (middle column), and grand-average deviant-minus-control difference waves and topographical maps of visual MMN (right column), in the three task-difficulty conditions (easy, moderate, and difficult). **(B)** Grand-average mean amplitudes of DRN, N1-difference, and visual MMN in the three task-difficulty conditions (electrode: PO8). Error bars indicate standard errors of the mean. **(C)** Grand-average peak latencies of DRN, N1-difference, and visual MMN in the three task-difficulty conditions (electrode: PO8). Error bars indicate standard errors of the mean with a jackknife method. Asterisks indicate a significant difference (*p* < 0.01).

Figure 3 shows the grand-average ERPs and EOGs in response to deviant, standard, and control stimuli in the easy (left column), moderate (middle column), and difficult conditions (right column). Figure 4A (left column) shows the traditional, grand-average deviant-minus-standard difference waves in the three task-difficulty conditions. A posterior negativity (DRN) that peaked at 188 ms (easy condition, PO8), 196 ms (moderate condition, PO8), and 203 ms (difficult condition, PO8) was observed. Figure 4A (middle column) shows the grand-average control-minus-standard difference waves in the three task-difficulty conditions. A posterior negativity (N1-difference) that peaked at 193 ms (easy condition, PO8), 196 ms (moderate condition, PO8), and 197 ms (difficult condition, PO8) was observed. Figure 4A (right column) shows the grand-average deviant-minus-control difference waves in the three task-difficulty conditions. A posterior negativity (visual MMN) that peaked at 186 ms (easy condition, PO8), 193 ms (moderate condition, PO8), and 225 ms (difficult condition, PO8) was observed; there was no clear sign of polarity-reversed N1.

### Scalp distributions of N1-difference and visual MMN

Figure 4A (middle and right columns) also shows topographical maps of N1-difference and visual MMN in the three task-difficulty conditions (within the 11-ms time-windows including ± 5 ms from the corresponding peak), respectively. The N1-difference had a scalp distribution that peaked at bilateral parieto-occipital electrodes (PO7 and PO8), while the visual MMN had a scalp distribution that peaked at a right parieto-occipital electrode (PO8), regardless of the task-difficulty condition. Three-Way ANOVAs (2 Difference waves × 13 Electrodes × 3 Task difficulties) performed on the mean amplitudes of difference waves (within the 11-ms time-windows including ± 5 ms from the corresponding peak) revealed significant main effects of Difference wave [*F*_(1, 21)_ = 12.96, *p* < 0.01, partial η^2^ = 0.38] and Electrode [*F*_(12, 252)_ = 26.21, *p* < 0.001, ε = 0.19, partial η^2^ = 0.56], as well as a significant interaction of Difference wave × Electrode [*F*_(12, 252)_ = 7.12, *p* < 0.001, ε = 0.25, partial η^2^ = 0.25]. Importantly, the significant interaction of Difference wave × Electrode was also present in the same Three-Way ANOVAs performed on the normalized mean amplitudes [*F*_(12, 252)_ = 4.28, *p* < 0.01, ε = 0.26, partial η^2^ = 0.17]. *Post-hoc* comparisons revealed that the interaction mainly arose from the fact that the scalp distribution of N1-difference was bi-lateralized, while that of visual MMN was more right-lateralized.

### Mean amplitudes of DRN, N1-difference, and visual MMN

Figure 4B shows the grand-average mean amplitudes of DRN, N1-difference, and visual MMN in the three task-difficulty conditions (within the 11-ms time-windows including ± 5 ms from the corresponding peak at PO8 electrode). For the DRN, the mean amplitude was −3.04 μV (*SE* = 0.34) in the easy condition, −3.14 μV (0.37) in the moderate condition, and −2.62 μV (0.23) in the difficult condition. One-tailed paired *t*-tests showed that DRN was significantly elicited in the easy [*t*_(21)_ = −8.79, *p* < 0.001, *d* = 1.87], moderate [*t*_(21)_ = −8.59, *p* < 0.001, *d* = 1.83], and difficult conditions [*t*_(21)_ = −11.24, *p* < 0.001, *d* = 2.39]. However, a One-Way ANOVA (3 Task difficulties) revealed no significant effect (*F* = 1.0). For the N1-difference, the mean amplitude was −1.90 μV (0.27) in the easy condition, −2.25 μV (0.29) in the moderate condition, and −2.15 μV (0.26) in the difficult condition. One-tailed paired *t*-tests showed that N1-difference was significantly elicited in the easy [*t*_(21)_ = −6.92, *p* < 0.001, *d* = 1.48], moderate [*t*_(21)_ = −7.80, *p* < 0.001, *d* = 1.66], and difficult conditions [*t*_(21)_ = −8.26, *p* < 0.001, *d* = 1.76]. However, a One-Way ANOVA (3 Task difficulties) revealed no significant effect (*F* = 1.2). For the visual MMN, the mean amplitude was −1.17 μV (0.33) in the easy condition, −0.89 μV (0.34) in the moderate condition, and −0.93 μV (0.31) in the difficult condition. One-tailed paired *t*-tests showed that visual MMN was significantly elicited in the easy [*t*_(21)_ = −3.56, *p* < 0.01, *d* = 0.76], moderate [*t*_(21)_ = −2.64, *p* < 0.05, *d* = 0.56], and difficult conditions [*t*_(21)_ = −2.97, *p* < 0.01, *d* = 0.63]. However, a One-Way ANOVA (3 Task difficulties) revealed no significant effect (*F* = 1.9).

### Peak latencies of DRN, N1-difference, and visual MMN

Peak latencies were calculated by a jackknife method (Miller et al., 1998; Ulrich and Miller, 2001; Kiesel et al., 2008). Figure 4C shows the grand-average peak latencies of DRN, N1-difference, and visual MMN in the three task-difficulty conditions (PO8 electrode). For the DRN, the peak latency was 188.7 ms (*SE* = 0.36) in the easy condition, 197.2 ms (0.25) in the moderate condition, and 203.5 ms (0.33) in the difficult condition. A One-Way ANOVA (3 Task difficulties) revealed no significant effect (*F*_corrected_ = 1.3). For the N1-difference, the peak latency was 194.3 ms (0.36) in the easy condition, 197.6 ms (0.16) in the moderate condition, and 197.9 ms (0.10) in the difficult condition. A One-Way ANOVA (3 Task difficulties) revealed no significant effect (*F*_corrected_ = 1.0). For the visual MMN, the peak latency was 185.9 ms (0.22) in the easy condition, 194.5 ms (0.81) in the moderate condition, and 226.2 ms (0.24) in the difficult condition. A One-Way ANOVA (3 Task difficulties) revealed a main effect of Task difficulty [*F*_corrected_(2, 42) = 4.35, *p* < 0.05, ε = 0.64, partial η^2^ = 0.17]. *Post-hoc* comparisons showed that the peak latency of visual MMN was longer in the difficult condition than in the easy condition [*t*_corrected_(21) = 5.59, *p* < 0.01].

### Mean amplitudes of visual evoked potentials

**Figure 5:**
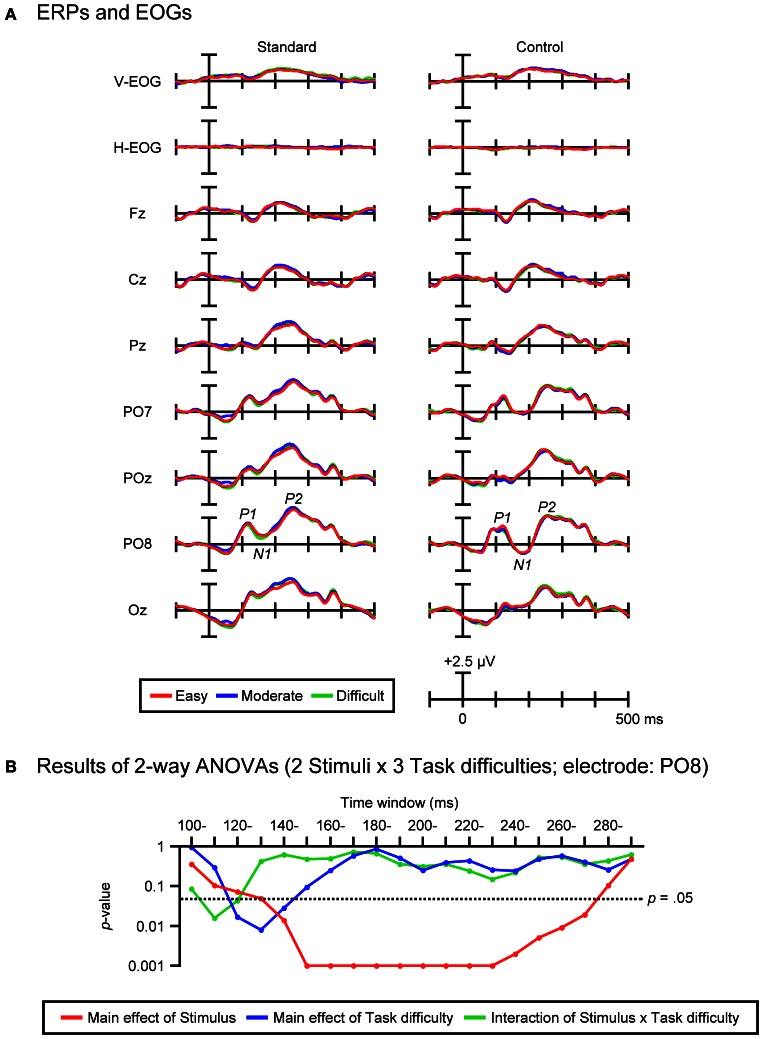
**(A)** Grand-average ERPs and EOGs in response to standard (left column) and control stimuli (right column) in the easy, moderate, and difficult conditions. **(B)** Results of Two-Way ANOVAs (2 Stimuli × 3 Task difficulties) performed on the mean amplitudes of standard and control ERPs within 20 consecutive 10-ms time-windows from 100 to 300 ms (electrode: PO8).

Figure 5A shows the grand-average ERPs and EOGs in response to standard (left column) and control stimuli (right column) in the three task-difficulty conditions. Figure 5B shows the results of Two-Way ANOVAs (2 Stimuli × 3 Task difficulties) that were performed on the mean amplitudes of ERPs elicited by standard and control stimuli (within each of 20 consecutive 10-ms time-windows from 100 to 300 ms). Reflecting the larger N1 in response to control stimuli compared to standard stimuli (see the control-minus-standard difference waves shown in the middle panel of Figure 4A), a significant main effect of Stimulus was revealed for the 14 consecutive 10-ms time-windows from 140 to 280 ms [*F*s_(1, 21)_ = 6.44–88.01, *p*s < 0.05–0.001, partial η^2^s = 0.24–0.81]. With regard to the Task-difficulty factor, a significant main effect of Task difficulty was revealed for the 3 consecutive 10-ms time-windows from 120 to 150 ms (i.e., the latency range of P1) [*F*s_(2, 42)_ = 4.04–5.68, *p*s < 0.05–0.001, εs = 0.93–0.96, partial η^2^s = 0.16–0.21]. *Post-hoc* comparisons showed that P1 elicited by both standard and control stimuli was smaller in the difficult condition than in the easy condition (*p*s < 0.05). Further, a significant interaction of Stimulus × Task difficulty was revealed for the 2 consecutive 10-ms time-windows from 110 to 130 ms (i.e., the latency range of P1) [*F*s_(2, 42)_ = 3.56–4.78, *p*s < 0.05, εs = 0.89–0.92, partial η^2^s = 0.15–0.19]. *Post-hoc* comparisons showed that P1 elicited by control stimuli was smaller in the difficult condition than in the easy condition (*p*s < 0.05), while P1 elicited by standard stimuli did not differ among the three task-difficulty conditions. Importantly, there was no significant main effect or interaction related to the Task-difficulty factor for the time-windows from 150 to 300 ms (i.e., the latency range of N1 and P2, where DRN, N1-difference, and visual MMN were emerged in the difference waves, see Figure 4A).

## Discussion

Previous studies have shown that DRN (most likely, consisting of visual MMN and N1-difference) is insensitive to the manipulation of task difficulty (Heslenfeld, 2003; Pazo-Alvarez et al., 2004), which supported the notion that visual MMN reflects attention-independent predictive processes. By taking into account the possible underestimation of the effect of task difficulty on visual MMN due to the superposition of N1-difference, we examined the effects of task difficulty on visual MMN, less contaminated by N1-difference, and investigated whether or not the predictive processes indexed by visual MMN are truly attention-independent.

### Effects of task difficulty on visual MMN

Behavioral performance in the size-change detection task for the central fixation circle deteriorated (i.e., reaction times became slower and hit rates decreased) as the task difficulty increased, although no significant difference was observed between the easy and moderate conditions. These results confirm that the difficulty of the size-change detection task was successfully manipulated by varying the magnitude of the size-change (at least there was a difference between the difficult condition and the other two conditions).

In traditional deviant-minus-standard difference waves, a posterior negativity was observed at around 100–300 ms (DRN). The latency and scalp distribution are highly similar to those of DRN observed in previous studies (see e.g., Pazo-Alvarez et al., 2003; Czigler, 2007; Kimura, 2012). The DRN was then decomposed into N1-difference and visual MMN. In control-minus-standard difference waves, a posterior negativity at around 100–300 ms with no clear hemispheric dominance (N1-difference) was observed, while in deviant-minus-control difference waves, a posterior negativity at around 150–300 ms with clear right hemispheric dominance (visual MMN) was observed; there was no clear sign of polarity-reversed N1 in deviant-minus-control difference waves. The latency and scalp distribution of these two components are similar to those of N1-difference and visual MMN observed in recent studies, respectively (e.g., Kimura et al., 2009, 2010b). The significantly different scalp distributions of these two components are also consistent with the recent finding that N1-difference evaluated in control-minus-standard difference waves and visual MMN evaluated in deviant-minus-control difference waves are generated from distinct cortical areas (Kimura et al., 2010a). Further, the clear right hemispheric dominance observed for the latter posterior negativity is a characteristic of visual MMN (e.g., Kimura et al., 2009, 2010b, 2012). These observations suggest that DRN would be decomposed into N1-difference reflecting refractory effects and visual MMN reflecting prediction error effects.^1^

Neither the mean amplitudes nor the peak latencies of DRN were affected by the task difficulty, which is consistent with previous studies which showed that task difficulty does not affect DRN (Heslenfeld, 2003; Pazo-Alvarez et al., 2004). Task difficulty also did not affect the mean amplitudes or peak latencies of N1-difference. This result implies that task difficulty did not significantly influence the refractoriness state of afferent neurons that engage in N1. Unlike the findings regarding DRN and N1-difference, while task difficulty did not affect the mean amplitudes of visual MMN, it did affect the peak latencies of visual MMN: the peak latencies were significantly delayed in the difficult condition compared to the easy condition. This result implies that, while task difficulty did not significantly influence visual MMN elicitation itself, it strongly influenced the speed (or efficiency) of visual MMN elicitation.

The delay of peak latencies of visual MMN is not attributable to the modulation of visual evoked potentials elicited by control stimuli as a function of task difficulty. The amplitudes of ERPs elicited by control as well as standard stimuli in the latency range of P1 were slightly smaller in the difficult condition than in the easy condition: 110–150 ms for the control stimuli and 130–150 ms for the standard stimuli. This result is consistent with previous studies which demonstrated that the amplitude of P1 is a reliable index of spatial attention allocation (Hillyard et al., 1995; Mangun and Hillyard, 1995; Hillyard and Anllo-Vento, 1998) and the amplitude of P1 elicited by task-irrelevant peripheral stimuli is reduced as the task difficulty is increased from easy to difficult, via decreasing the amount of spatial attention allocated to the task-irrelevant peripheral stimuli (Handy and Mangun, 2000; Handy et al., 2001). Importantly, unlike the amplitudes of ERPs in the P1 latency range, those of ERPs in the subsequent N1 and P2 latency range were not affected by the manipulation of task difficulty for both control and standard stimuli: 150–300 ms, including the latency range of visual MMN as well as DRN and N1-difference in the difference waves. This result ensures that the delayed peak latency of the posterior negativity in the deviant-minus-control difference waves truly represents the modulation of visual MMN elicited by deviant stimuli.

The result that the peak latencies of visual MMN were delayed with an increase in the task difficulty is compatible with the expectation from the perceptual load theory (Lavie and Tsal, 1994; Lavie, 1995, 2005). This theory proposed that, as the perceptual load of task-relevant central information increases, a greater portion of the attention resources is needed for the perceptual processing of this information, and as a result, fewer residual attention resources are available to be involuntarily allocated for the perceptual processing of task-irrelevant peripheral information. The present effect of task difficulty on visual MMN can be interpreted as follows: as the difficulty of the size-change detection task increased from easy to difficult, a greater portion of the attention resources became necessary for detection of the size-change and fewer residual attention resources became involuntarily allocated to task-irrelevant surrounding bar stimuli, which caused the less rapid (less efficient) elicitation of visual MMN.

The present findings may be in line with a recent finding that visual MMN in response to task-irrelevant deviation is sensitive to the congruency between the feature dimension of task-irrelevant deviation and that of task-relevant target (Czigler and Sulykos, 2010). In that study, the authors presented task-irrelevant oddball sequences consisting of deviant and standard bar stimuli with either different colors or different orientations at the peripheral visual fields in separate blocks while the participant performed either a color- or an orientation-change detection task regarding a continuously presented shape at the central visual field in separate blocks. They found reduced amplitudes and delayed peak latencies for visual MMN in response to deviant stimuli when the feature dimensions of deviation and target were congruent (e.g., color deviant stimuli under a color-change detection task) compared to when they were incongruent (e.g., color deviant stimuli under an orientation-change detection task). They interpreted the reduction of amplitudes and delay of peak latencies of visual MMN in terms of the competition for feature-specific attentional resources (Desimone and Duncan, 1995): when congruent, the processing of task-relevant target and task-irrelevant deviation compete for feature-specific attentional resources, which leads to the suppression of visual MMN in response to the task-irrelevant deviation. More interestingly, although they did not consider the effects of task difficulty on visual MMN, as in the present study, their results showed delayed peak latencies of visual MMN for the difficult task (i.e., the color-change detection task) relative to the easy task (i.e., the orientation-change detection task) (however, their study evaluated visual MMN in deviant-minus-standard difference waves, and thus it is possible that the delayed peak latency may represent the modulation of N1-difference). Although the experimental and analysis procedures differed between the present study and that reported by Czigler and Sulykos (2010), in a broad context, the findings in these studies consistently shed new light on the attention-sensitive nature of visual MMN (for another example, see Kimura et al., 2010d; but see also Winkler et al., 2005; Berti, 2011, for contrasting examples).

In summary, we found that visual MMN can be affected by the manipulation of task difficulty. This result suggests that visual MMN is not necessarily insensitive to the amount of attentional allocation. In contrast to the previous understanding, the present study supports the notion that visual MMN involves attention-demanding predictive processes.

### Theoretical and practical implications

The present findings suggest that at least some portion of predictive processes underlying visual MMN elicitation is attention-demanding. According to the predictive framework of visual MMN (Kimura et al., 2011; Kimura, 2012), the elicitation of visual MMN requires the contribution of multiple predictive processes: (1) the extraction of sequential rules embedded in the temporal structure of successive visual stimulation, (2) the establishment of a predictive model that encodes the extracted sequential rules, (3) the formation of a temporally-aligned prediction about forthcoming visual events on the basis of the predictive model, and (4) the comparison of the current and predicted visual events. Visual MMN is the output of these predictive processes: when incongruence has been detected via the comparison, visual MMN emerges. According to this framework, the delay of visual MMN elicitation observed in the present study implies that the comparison process required more time as the task difficulty increased from easy to difficult. At present, it is difficult to determine whether the delay represents the direct influence of attention on the comparison process (cf. Berti, 2011) or is the result of attentional influence on processes earlier than the comparison process (cf. Kimura et al., 2010b,c), providing no clue as to which part of the predictive process is attention-demanding. Determination of the attention-sensitivity of each process should be an important challenge in future visual MMN research, which could lead to the establishment of an integrative theory of sensory prediction and attention.

Research in this area should be important not only for theoretical development but also for practical progress. To date, visual MMN has been used in clinical studies as an effective tool for investigating preattentive visual processing, and has shed new light on its abnormality in the elderly (e.g., Tales et al., 2002) and several clinical populations (e.g., Tales and Butler, 2006; Tales et al., 2008; Urban et al., 2008; Chang et al., 2011; Qiu et al., 2011). With regard to such clinical applications, the present findings offer two implications. First, although visual MMN can be reliably regarded as a reflection of automatic visual processing (in that the elicitation of visual MMN does not require attention to be actively directed to visual stimulation), it can no longer be regarded as a reflection of preattentive visual processing (in that not all of the predictive processes that underlie visual MMN elicitation can be considered to be attention-independent). Second, possible attentional influences on visual MMN should always be taken into account: significant between-group differences in visual MMN may represent differences in automatic visual processing itself or may represent differences in attentional influences on automatic visual processing. A better understanding of the attention-sensitivity of the aforementioned predictive processes would be helpful for optimizing experimental design, so that such an ambiguous interpretation can be avoided.

Finally, the present findings suggest that visual MMN may be an effective tool in ergonomics (human factors) studies. In this research field, there has been a substantial interest in the utility of ERPs for the assessment of mental workload in the laboratory or real-world tasks (Donchin et al., 1986; Kramer and Weber, 2000). One of the major ERP procedures for assessing the mental workload is the so-called “probe” technique. In this procedure, while the participant performs a certain primary task, stimuli that are unrelated to the primary task (i.e., probe stimuli) are presented concurrently. To date, it has been suggested that P300, sensory evoked potentials, or other ERPs in response to probe stimuli can be used to assess the mental workload in the primary task (e.g., Kramer et al., 1983, 1995; Wickens et al., 1983; Ullsperger et al., 2001). Although the conditions for the application of visual probe stimuli would be fairly limited compared to those for the application of auditory or somatosensory probe stimuli, the utility of visual MMN in ergonomics applications may deserve more attention, given the unique (automatic but still attention-sensitive) nature of visual MMN.

## Conclusions

The present study demonstrated that visual MMN can be affected by the manipulation of task difficulty, which suggests that visual MMN is sensitive to the amount of attentional allocation. In contrast to the previous understanding, the present finding supports the notion that visual MMN involves attention-demanding predictive processes.

### Conflict of interest statement

The authors declare that the research was conducted in the absence of any commercial or financial relationships that could be construed as a potential conflict of interest.

## Footnotes

^1^One may argue that the posterior negativity extracted in the deviant-minus-control difference waves may still include N1-difference, since the smallest physical separation between control stimuli (ca. 16.4°) was smaller than the physical separation between deviant and standard stimuli (ca. 32.7°), and thus, the state of refractoriness for control stimuli might be higher than that for deviant stimuli. To rule out this possibility, we calculated ERPs elicited by control stimuli that were preceded by other control stimuli with at least ca. 32.7° physical separation. We found that, even with the newly-calculated control ERPs, the same statistically significant pattern of results regarding the visual MMN and N1-difference were obtained. This indicates that the present equiprobable protocol was sufficient for keeping the state of refractoriness for control stimuli equal to (or even lower than) that for deviant stimuli.
